# Knowledge, Perception and Practice towards the Risks of Excessive Weight Gain during Pregnancy among Pregnant Mothers at Myung Sung Christian Medical General Hospital, Addis Ababa, Ethiopia

**DOI:** 10.4314/ejhs.v31i2.20

**Published:** 2021-03

**Authors:** Mahlet Alebachew, Amarech Doyo, Desta Admasu, Kokeb Sisay, Tariku Shimels

**Affiliations:** 1 Kea Med Medical College, Department of Nursing; 2 St. Paul's Hospital Millennium Medical College, Research Directorate

**Keywords:** Excessive weight gain, BMI, overweight, obese, knowledge, perception

## Abstract

**Background:**

Being overweight and obese represents a severe public health deterioration affecting all population in general and vulnerable groups, such as pregnant women in particular. This study aimed to assess the knowledge, perception and practice towards the risks of excessive weight gain during pregnancy among pregnant mothers at Myung Sung Christian Medical General Hospital.

**Methods:**

An institution based cross-sectional study was conducted from July to August, 2019. A systematic random sampling technique was employed to select participants. A total of 176 respondents were included in the study. Data was collected using interviewer administered questionnaire, observation checklist, and measurements of weight and height. Statistical product and service solution version 20.0 was employed for analysis. Descriptive statistics, using tables and charts, was used to present results.

**Results:**

Gestational diabetes mellitus (83.5%) and high blood pressure (80.7%) were the major known risks associated with excessive weight gain during pregnancy. Based on the mean score, 96(54.5%) had poor knowledge while 80(45.5%) had good knowledge about the risks of excessive weight gain. The majority (92.0%) overweight and all obese pregnant mothers did not know their actual weight status. Similarly, 134(76.1%) of the study participants were not engaged in regular physical exercise during the current pregnancy.

**Conclusion:**

This study revealed that pregnant mothers attending in Korean General Hospital were generally poorly knowledgeable on the risks of excessive weight gain during pregnancy, had poor perception on current weight status and poor practices on their weight gain management. Facility based education and community awareness creation should accompany antenatal care services.

## Introduction

Overweight and obesity, defined as a body mass index (BMI = weight [kg] / height^2^ [m^2^]) of 25 – 29.9 and >30kg/m^2^ respectively, describe body size whereby ‘abnormal or excessive fat accumulation may impair health (1). It represents a severe public health problem, mainly due to the global trend towards increased prevalence rates and the impact it bears on productivity of society (1,2).

According to the World Health Organization (WHO), more than 1.9 billion adults are overweight (OW), including over 600 million men and women considered obese (OB) (1,3). Paralleling this trend of increased prevalence of excess weight in the general population, the number of OW or OB pregnant women presenting for obstetric care continues to grow worldwide (4). While OW or OB bears devastating complications and outcome when combined with pregnancy, there is a considerable negligence among those at risk to prevent or control it with recommended strategies before or during pregnancies (5).

In Africa, despite a high prevalence of under nutrition, the prevalence of OW is increasing at an alarming rate. The most recent surveys among urban women, Egypt has the highest prevalence of OW (44%) and OB (39%) by far, followed by Ghana with an OW prevalence of 30% and obesity of 22% of urban women (6).

In Ethiopia, the urban prevalence of OW among women was reported to be 12.5% and OB as 3.6% (10), whereas the 2016 Ethiopia demographic health survey (EDHS) reported that since 2000, OW or OB has increased from 3% to 8% in 2016 (7). In Ethiopia, recently conducted studies have shown that the prevalence of excessive weight gain among pregnant women ranged from 2.7% in Harari (8) to 6.1% in Addis Ababa (9).

Gaining too much weight during pregnancy can produce immediate and long-term health risks to a woman and her infant (10). An elevated pre-pregnancy BMI was reported to be linked to depression (11), preeclampsia, hypertension, gestational diabetes and birth defects (12) while reports in the first trimester showed its association with outcomes, such as induction of labor, caesarian section (13) and higher odds macrosomia (14). Likely, findings from third trimester pregnancy reveal that raised BMI was associated with higher chorioamnionitis and cesarean delivery rates, higher neonatal birth weight, and ponderal index (15). Further, it was suggested to record both weight and height of pregnant mothers as BMI may be predictive of gestational hypertension, preeclampsia, gestational diabetes, large for gestational age neonate at later trimesters (16). On the other hand, though inadequate weight gain poses little health risk to mothers, it may result in small growth-restricted infants, which increases the risk for infant mortality and developmental delay (14,17,18).

While the adverse outcomes of, especially excessive weight gain are apparent, education and practices for intervention are yet minimal. Guidelines from both the American College of Obstetricians and Gynecologists (17) and the American Dietetics Association (19) recommend that women who are OW or OB be counseled prior to conception and encouraged to adopt lifestyle changes to minimize the risk of developing complications during pregnancy related to being OW or OB. For many women, this is not easily achievable as it is estimated that excess of 50% of the pregnancies are unplanned (18). Therefore, the focus moves to minimizing risk during pregnancy via restriction of GWG (6,7,18).

Identifying existing knowledge, perception and practice gaps and creating awareness can produce a profound effect on mothers' realization of gestational overweight and obesity. If women do not understand their own BMI, they may not recognize the need for weight loss intervention (20). Studies showed that a greater proportion of women knew the risks of being OW on gestational diabetes mellitus (GDM), high blood pressure (BP), pre-eclampsia, cesarean section, and still birth (7,21). It was also reported that about 19% of pregnant women have poor overall knowledge, and among those who are OW, only 30% are reported to identify themselves correctly (21). On the contrary, OW women underestimated their BMI while underweight women did the opposite (22).

Despite availability of reports on dietary habits and practices (23) during pregnancy and disfavor of weight gain in the rural communities (24), the status of knowledge, perception and practice towards the risks of excessive weight gain, in urban settings, is scarcely known. The objective of this study was to estimate the level of knowledge, perception and practice towards excessive weight gain during pregnancy among pregnant women attending at a private general hospital in Addis Ababa, Ethiopia.

## Methods

**Study setting**: The study setting, Myung Sung Christian Medical (MCM) General Hospital (commonly called as Korean hospital) is found in Addis Ababa City, Bole Sub City. The hospital was established in 2004 by the Myung Sung Presbyterian Church of Korea. The following services are provided by the MCM general hospital: emergency services, delivery, other obstetrics and gynecology services, minor and major operation services, dental and eye treatment services, laboratory, pharmacy, intensive care unit (ICU), neonatal intensive care unit (NICU), ultrasound, magnetic resonance imaging (MRI) radiology services, inpatient and outpatient services including antenatal care (ANC) follow-up of mothers. During the study period, around 300 pregnant mothers were at follow-up.

**Study design and period**: An institution based cross-sectional study was conducted from 1^st^ July to 1^st^ August, 2019.

**Source population**: All pregnant women who visited ANC clinic of MCM General Hospital in Addis Ababa, Ethiopia

**Study population**: Pregnant women who visited the ANC clinic of MCM General Hospital during the study period and fulfilled the inclusion criteria

**Sample size**: The sample size for the study was estimated using the single population proportion formula. To the best of our knowledge, quantitative scientific report on knowledge and perception about the risks of excessive weight gain during pregnancy among pregnant mothers in Ethiopia was scanty. Accordingly, it was computed using P =50%, Z =1.96 (for 95% level of confidence), and D= 0.05 (for allowed margin of error). Since the source population was less than 10,000, a final population correction formula was applied and adding 5% for non-response rate, the final sample included in the study was 176 participants.

**Sampling technique**: The medical record number (MRN) of pregnant mothers was used as a sampling frame. A systematic random sampling technique was applied to select potential participants using their ANC identified registration number and appointments. Out of the total 300 participants expected to have ANC visits during the study period, 110 were selected systematically assigned with every odd MRN (drawn randomly), and 66 were selected consecutively until the final sample size was reached. In cases a respondent was not eligible, the immediate next respondent was considered.

**Inclusion criteria**: Pregnant women who already had started their ANC visit at least once during their current pregnancy and had willingness to participate in the study were included.

**Exclusion criteria**: Pregnant women with confirmed twin or more pregnancy were excluded. In addition, pregnant women who were too ill to participate in the interview and those with first visit to the ANC were excluded from the study.

**Data collection instrument and process**: Data was collected using a self-administered structured questionnaire, a checklist and measurement of participant characteristics. The questionnaire was developed by adapting from previous similar studies and reviewing of different literature (8,9,21,25). The sociodemographic profiles, knowledge, perception and practices of participants were collected using the self-administered questionnaire to pregnant mothers. ANC visit and current medical condition were checked from patient charts. Data on weight and height of the mothers was measured in standardized measuring tape and weight scale at the time of data collection by the investigators.

**Data quality management**: Data was collected and cross-checked by the team of investigators. To assure the quality of the data collected, the questionnaire were first prepared in English language and then translated into Amharic (the local official language) and the Amharic version was translated back to English to make it clear, complete and consistent during entry and analysis. A pretest was done on 5% of the required sample in a similar setting. Questions which posed difficulty and became unclear were rephrased and corrected, and unnecessary questions were excluded after pretest. To ensure content validity, the instrument was reviewed and checked by experts in the field.

**Ethical clearance**: Ethical clearance was obtained from the Research and Ethics Committee of Kea Med College, Department of Nursing. Permission was obtained from MCM General Hospital to conduct the study. The purpose of the study was clearly explained to the participants, and informed consent was obtained from each study participant. Participants were also informed their full right to withdraw or refuse to participate in the study. Data was analyzed in aggregate and confidentiality of the data collected was maintained.

**Data analysis**: The data from the questionnaire was checked manually for completeness, coded, entered in statistical product and service solution (SPSS) version 20.0 and, checked and cleaned again before analysis. Descriptive statistics was applied to analyze the collected data. The results of the study are presented in tables and charts.

***Operational definition***

**Knowledge**: Understanding the risks of excessive weight gain during pregnancy was measured based on score of knowledge questions. Good knowledge is a score that fell on and above the mean while knowledge score below the mean was considered as poor knowledge.

**Perception**: A pregnant mother's own conception about considering herself either as underweight, normal weight, overweight or obese. Accordingly, when a pregnant mother couldn't identify the current actual weight status, it was considered as poor perception and vice versa.

**Practice**: It is measured based on a mother's engagement in either regular exercise or dietary habits for weight management and healthy life. Regular exercise was assumed any usual activity (including house work) and walking of any duration a pregnant woman reported to have had during the current pregnancy, and measured in weekly frequency. Likely, dietary practice was assumed based on a pregnant woman's dietary intake report in terms of frequency and diversification during the current pregnancy.

**Diversified meal**: In the present study, variety of foods is considered as a dietary intake which consists of carbohydrates, proteins, fat, vegetables and fruits reported by participants.

## Results

**Socio demographic characteristics of the study participants**: In this study, a total of 176 study participants were included. The majority of the study participants were above 30 years old accounting about 78(44.3%) with a mean (SD) age of 29.86 (±3.99) years, married 78(89.8%) and with education level of above 12^th^ grade 147(83.5%) (Table 1).

**Table 1:** Socio-demographic characteristics of pregnant women visiting ANC clinic at MCM general hospital, August 2019 (n=176)

Characteristics	Value	Frequency	Percentage (%)
**Age (Years)**	21–25	34	19.3
	26–30	64	36.4
	>30	78	44.3
**Marital status**	Single	7	4
	Married	158	89.8
	Widowed	1	0.6
	Divorced	10	5.7
**Education Level**	Primary	10	5.7
	Secondary	19	10.8
	Above 12	147	83.5
**Employment Status**	Merchant	24	13.6
	Housewife	45	25.6
	Student	1	0.6
	Government employee	28	15.9
	Private employee	78	44.3
**Religion**	Orthodox Christian	74	42.0
	Protestant	56	31.8
	Muslim	39	22.2
	Others*	7	4.0
**Income**	Do not know	38	21.6
	<3000	34	19.3
	3001–5000	70	42
	Above 5000	30	17

Others*: refers to Catholic and Jehovah Witness. Income classification was made by considering the Ethiopian civil servants' salary scale, the income distribution reported by participants and literature

**Knowledge about risks of excessive weight gain during pregnancy**: Among 176 study participants, 128(72.7%) had source of information about the risks of excessive weight gain during pregnancy. Of these, 58(45%) obtain from family, 41(32%) from friends, 16(12.5%) from media, 8(6.3%) from a health professional and 5 (3.9%) from all sources.

The knowledge of respondents about the risks of excessive weight gain during pregnancy was determined using 10 knowledge questions. Participants were asked to identify from a list of 10 response options (i.e. GDM, hypertension, infection, hemorrhage, stillbirth, SGA, macrosomia, fetal structural anomaly, caesarean delivery and miscarriage) which each was a risk due to excessive weight gain during pregnancy. Participants received one point for each response option they endorsed. Total scores ranged from 0 to 10 scores. Based on their mean score responses, the respondents were grouped as having either good knowledge or poor knowledge about risks of excess weight gain during pregnancy.

**Figure 1:**
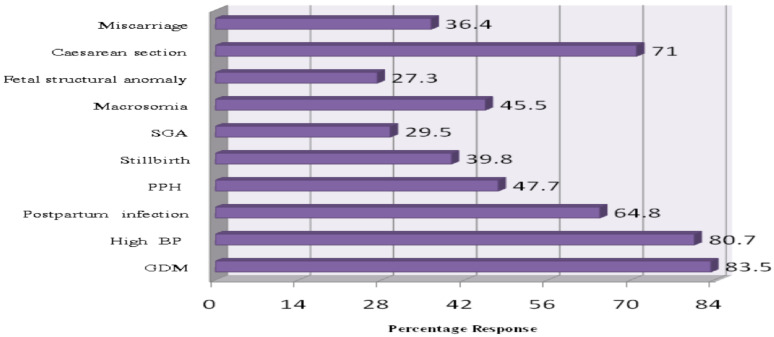
Knowledge of participants on the risks excessive weight gain during pregnancy among pregnant mothers visiting ANC clinic at MCM general hospital, August 2019 (n=176)

The majority of the study participants knew gestational diabetic mellitus (GDM) (83.5%), high BP (80.7%), and caesarian section (71.0%) could be risks associated with excessive weight during pregnancy (Figure 1). Looking into overall knowledge assessment, the mean (±SD) knowledge score of study subjects was 5.26 (±2.76) with a minimum score 0 and maximum score of 10. Based on the mean, 96(54.5%) of the participants had poor knowledge and 80(45.5%) had good knowledge about the risks of excessive weight gain during pregnancy.

**Figure 2:**
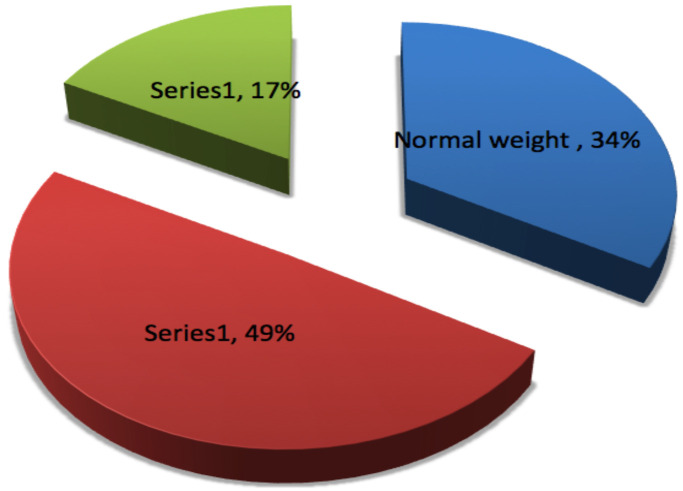
BMI of pregnant mothers visiting ANC clinic at MCM general hospital, August 2019 (n=176).

**BMI status of pregnant women visiting the ANC during the study period**: Body mass index (BMI) of study participants was computed by dividing the weight, in kilogram, of a pregnant woman to her height in meters. The current BMI mean (±SD) of the study participants was 26.5 Kg/m^2^ (±3.66 Kg/m^2^) with a minimum of 19 Kg/m^2^ and maximum of 39.2 Kg/m^2^. Of the 176 participants, 87(49%) pregnant women were classified as overweight (BMI 25–29.9 Kg/m^2^) whereas 59(34%) were classified as normal weight (BMI 18.5–24.9 Kg/m^2^), and 30 (17.0%) were classified as obese (BMI ≥30) (Figure 2).

**Perception of pregnant mothers towards the current weight status**: Pregnant mothers' perceptions towards their weight status are presented in Table 2, which were determined by asking participants, “At which of the following weight category are you considered (perceive) yourself?” Response options were underweight, normal weight, overweight or obese. Among 59 study participants who were actually normal weight, 54(91.5%) correctly perceived themselves. However, 5(8.5%) normal weight study participants incorrectly perceived themselves as they were under weight. From 87 study participants who were actually overweight, 80(92.0%) incorrectly perceived themselves as being normal weight. Similarly, from 30 actually obese pregnant mothers, none correctly perceived themselves as obese.

**Table 2:** Pregnant mothers' perception towards their own weight level at ANC clinic of MCM general hospital, August 2019 (n=176)

	Perceived Under weight	Perceived Normal weight	Perceived Over weight
Normal weight =59	5(8.5%)	54(91.5%)	0(0.0%)
Over weight =87	2(2.3%)	80(92.0%)	5(5.7%)
Obese =30	0(0.0%)	24(80.0%)	6(20.0%)

**Practice of the study participants towards weight gain management during pregnancy**: Regarding on regular physical exercise for weight gain management during their pregnancy, 134(76.1%) of the study participants were not engaged in regular physical exercise. Among 42 pregnant women who were engaged in regular physical exercise, the majority, 32(76.2%), had physical exercises once per week, 9(21.4%) had physical exercises two to three times per week and only one (2.4%) pregnant mother had physical excises more than three times per week.

**Figure 3:**
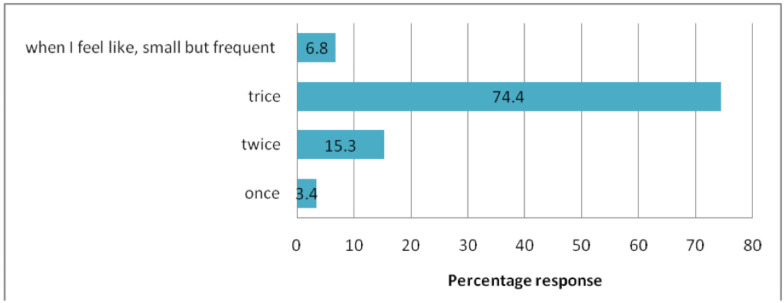
Meal frequency of pregnant mothers visiting ANC clinic at MCM general hospital, August 2019 (n=176).

Figure 3 below shows participants' responses on their nutritional practices during pregnancy. About a three forth (74.4%) of the respondents eat three times a day while 6(3.4%) eat once per day. More than half (59.7%) of the respondents claimed that they ate a variety of foods in moderation, while 33(18.8%) ate mostly carbohydrate-rich meals. In addition, 101(57.4%) respondents ate fruits and vegetables daily while 24 (13.6%) respondents ate fruits and vegetables once a week. Only 6(4%) of the study participants drank alcohol in the current pregnancy.

## Discussion

The present study showed that the majority of the participants on the assessment of knowledge scored high about GDM, high BP, and caesarean section to be the perinatal complications associated with excessive weight gain during pregnancy. These consequences are quite often reported in the literature too, for a link with gestational weight (26). The observed good awareness could, possibly, be due to growing prenatal service coverage which, in turn, might have led to exchange of maternal education during follow-up visits. Such maternal health alarms are also widely known by families and friends in urban areas. This finding is replicated in a similar study done on central Georgia on knowledge and perception of risks and complications of maternal obesity during pregnancy (21).

However, it has been found that respondents' knowledge about the risks of GWG on stillbirth, miscarriage, SAG and fetal structural anomaly was poor. Since these issues pertain to medical knowledge, the imprecise level of understanding can be attributed to the fact that the actual source of information for the majority was families, friends and media. Even though healthcare providers are trustable and credible sources of information, only 6.3% of the respondents heard from health professionals. This shows that despite the high perinatal care (PNC) services in Ethiopia, there still remains a limitation to properly address knowledge gap of pregnant women on overweight pregnancy complications. It was reported, elsewhere, that a socio-ecological dimension of determinants including personal challenges, provider related and, health system/program could be important to understand barriers and facilitators of PNC in women (27). A qualitative study on women's perceptions and discussions about gestational weight gain with healthcare providers during pregnancy also reflected that communication with healthcare providers about gestational weight gain was lacking apart from recording weight on charts (28).

The overall knowledge about the risks of excessive weight gain during pregnancy revealed that more than half of the study participants had low awareness of the perinatal complications associated with excessive weight gain. Yet, greater attribution for this gap can be derived from their source of advice as most do not get direct information from healthcare providers. A recent study reported that clinical and interpersonal care processes are most essential to quality PNC service which keep women engaged in the care and build trust about their care giver (29). Literature also shows that women may be more aware of personal long - term health risks rather than of perinatal risks associated with obesity (21,22). Better awareness of these complications may provide a motivating factor for women to maintain appropriate weight and practice of healthy life styles (30).

When we look at the self-perception of respondents about their own current weight status and compare it to their actual weight, most of the normal weight study participants correctly perceived themselves as they are normal weight. However, nearly all (92%) of the overweight and 100% of the obese study participants incorrectly perceived themselves as they were being normal weight. This finding was also in agreement with other studies where overweight and obese women had significantly higher percentage of error in self-identity (21). Studies among non-pregnant women also showed that BMI was associated with high odds of misperception at normal weight (31) and both at normal and overweight (32). This may reiterate the importance of pre-conception health education and awareness creation during perinatal follow-ups.

Studies (30,33) reported that physical exercise during pregnancy is associated with important benefits, including less gestational weight gain, lower incidences of GDM, GHTN, gestational hypertension disorders, preterm birth, and cesarean delivery, lower birth weight, and higher incidence of vaginal delivery. More than thee forth of the participants, in the present study, were not engaged in regular physical exercise. This may be due to lack of knowledge on the importance of exercise during pregnancy. Exercise counseling by medical personnel was also recommended to enhance women's involvement in exercise plans (34). This was similar with a study conducted on educational brochures influence on beliefs and knowledge regarding exercise during pregnancy (33). Generally, however, physical inactivity is more prevalent among women than men (35) and no adequate attention is paid to women's engagement in exercise (36). Alleviating gender and socio-ecological related barriers has been suggested to improve women's involvement in regular physical exercise (37).

Although more than half of the respondents claimed that they ate variety of foods in moderation, only 6.8% respondents ate whenever they felt like small, but frequently. There is a guideline (5) that encourages pregnant mothers to eat small but frequent meals. This finding is inconsistent with the study done in Nigeria where 52.9% of the study participants ate whenever they felt like (38). Nonetheless, the proportion of women who obtained good dietary practice in the present study is higher than figures reported in other parts of the country (39). Socio-economic variations such as family income, information source and perceived knowledge on importance of good dietary practice could account for a profound source of variation between the reports.

In this study, quite a few of the study participants drank alcohol in current pregnancy (4%). Although this figure is lower than similar measures in the literature (40), use of alcohol is associated with the risk of birth defects, especially, when consumed during the first trimester of pregnancy (41). Babies born to alcoholic mothers may develop fetal alcohol syndrome (42), which is characterized by low birth weight, and slow growth and development. In some cases, there may be permanent mental retardation.

The study is narrow to produce generalizable estimates and was limited only to descriptive presentation. However, it has provided baseline information on the level of knowledge, perception and practice of pregnant mothers towards the risks of excessive weight gain. The findings are, thus, supposed to inform maternal healthcare interventions be comprehensive and practical to managing weight gain during pregnancy. Future studies with prospective and multicenter approaches are warranted to produce reliable evidence.

In general, this study revealed that pregnant mothers attending in MCM General Hospital were generally poorly knowledgeable on the risks associated with excessive weight gain during pregnancy, had poor perception on current weight status and were poor in practicing their weight management. Hospital healthcare providers should establish proper client-provider relationship to build trust during PNC. Women should also be advised on appropriate preconception as well as gestational weight gain, promote physical activity and frequent small meals. Future research on prospective designs is warranted to produce more reliable findings and identify the risk factors.
